# Valence and interactions in judicial voting

**DOI:** 10.1098/rsta.2023.0140

**Published:** 2024-04-15

**Authors:** Edward D. Lee, George T. Cantwell

**Affiliations:** ^1^ Complexity Science Hub Vienna, Josefstædter Strasse 39, Vienna, Austria; ^2^ Department of Engineering, University of Cambridge, Cambridge CB2 1PZ, UK; ^3^ Santa Fe Institute, 1399 Hyde Park Road, Santa Fe, NM, USA

**Keywords:** maximum entropy, voting, US Supreme Court, bias, statistical inference, group interactions

## Abstract

The collective statistics of voting on judicial courts present hints about their inner workings. Many approaches for studying these statistics, however, assume that judges’ decisions are conditionally independent: a judge reaches a decision based on the case at hand and his or her personal views. In reality, judges interact. We develop a minimal model that accounts for judge bias, depending on the context of the case, and peer interaction. We apply the model to voting data from the US Supreme Court. We find strong evidence that interaction is an important factor across natural courts from 1946 to 2021. We also find that, after accounting for interaction, the recovered biases differ from highly cited ideological scores. Our method exemplifies how physics and complexity-inspired modelling can drive the development of theoretical models and improved measures for political voting.

This article is part of the theme issue ‘A complexity science approach to law and governance’.

## 1. Introduction

Judges are, in principle, impartial referees that adjudicate between opposing sides on the basis of the law and the substance of the case. But no judicial decision occurs in a political vacuum. Judges are buffeted by exogenous and endogenous forces that leave statistical traces in their voting patterns. One way to identify these forces or biases would be to regress the outcomes of votes against the valence or context of the decision. In the context of US politics, for example, a judge who votes consistently with a liberal or conservative valence could be considered to hold the corresponding viewpoint [1], but a liberal-conservative valence is only one choice. Alternatively, one could look to analyse decisions by attributing valences that correspond to espousing certain legal theories [2], the legitimacy of the court [3], adherence to and against group norms [4,5] or expression of physical disposition [6–9]. Several hypotheses have been explored to unpack judge impartiality [10,11]. Still, it is challenging to rigorously select between the myriad effects that may or may not sway judges’ collective decision-making [12].

How are we to determine the relevant context for any particular decision? Even if were able to divine context, consistently coding the voting outcomes with the valence is non-trivial and a source of debate among experts [13]; after all, the process of labelling introduces epistemological biases. Nevertheless, labelling decisions based on ideology or issue can be an important step for voting analysis [14] and such labelling is often assumed as a given for axiomatic voting models [15,16]. In this article, we propose a variation on minimal voting models that allows us to address this problem. The approach does not explicitly posit the context in which judges render decisions and instead allows for it to be discovered in the data. This permits us to recover the effects of individual bias while, and in constrast to issue-space models, explicitly measuring the effect of peer influence in each context.

We develop our approach for judicial voting using data from the US Supreme Court. The court is composed of a maximum of nine judges, nominated by the President and confirmed by the Senate, and decisions are reached through majority consensus. The court is adversarial: the justices adjudicate between two opposing sides (e.g. plaintiff and defendant, appellant and appellee). Thus, we can encode the vote of each judge in a binary fashion, denoting the vote of judge i as $s_{i}=+1$ or $s_{i}=-1$, where the sign indicates the party for which the judge voted. Of course, the particular assignment of the + and − sign to each party is not intrinsically meaningful [17]. We could equally well assign +1 as a vote in favour of the plaintiff as we could the defendant. To account for arbitrary nature of the assignment, any probabilistic model of voting should assign equal probability to a vote of +1 or −1, i.e. the probability for judge i’s vote should obey $P(s_{i}=+1)=P(s_{i}=-1)=1/2$. Throughout this paper, we focus on two natural courts (periods of time with unchanging membership): the Second Rehnquist Court (1994–2005) with K=922 votes and a Roberts Court (2020–2021) with K=114 votes. The two serve as base examples for comparing the approach on courts with differing ideological composition.

Our analyses recover effects of both consensus and division in the courts and individual biases in line with dominant perceptions of ideology but with differences from widely used models for attributing ideology. In addition, we find strong quantitative evidence of peer interactions despite having accounted for individual bias, which we show can lead to significant correlations. Such interactions are typically not included in voting models meant to extract individual biases in political science or legal studies [18,19], and so they are obscured by modelling assumptions. Overall, we demonstrate a context-sensitive and minimal approach to voting that incorporates both individual biases and peer-influence, while also balancing model complexity. This framework is a powerful way of testing quantitative hypotheses about voting behaviour.

In §1, we define the models we consider. We discuss how to fit these models to data, accounting for their complexity. In §2, we apply our methods to data from the US Supreme Court. Finally, we discuss the implications of our findings in the discussion.

## 2. Models

### (a) Voter bias with conditional independence

In the simplest model of voting behaviour, the judges would act independently of one another. The vote of a judge on a randomly selected case can be modelled as the flip of a biased coin. The joint probability P(s) of seeing the vector $s=(s_{1},\ldots ,s_{n})$ of votes for each judge 1 to n, is the product of each judge i’s probability $P(s_{i})$,

2.1 $$P(s)=\prod_{i=1}^{n}P(s_{i}).$$

Without prior knowledge of the context, a vote for either party could as well have been mapped to +1 as to −1, and the average vote must be

2.2 $$E[s_{i}]\equiv \sum_{s}s_{i}\;P(s)=0.$$

Equation (2.2) reflects the fact that if the votes are not coded (i.e. to give particular significance to +1 versus −1), the pattern of votes by any individual provides no information about their bias.

While the average votes may be uninformative, the correlations may be highly informative. A simple metric to consider is the covariance

2.3 $$C_{ij}=E[s_{i}s_{j}]-E[s_{i}]E[s_{j}].$$

**Figure 1.**
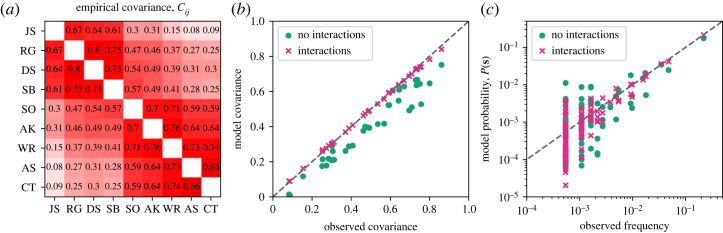
(*a*) Empirical covariance between judges on the Second Rehnquist Court. All judges are positively correlated. (*b*) Observed covariance compared to those predicted by models that ignore or incorporate interactions. (*c*) Observed frequency of votes against model predicted probability of votes. Judge initials are JS for John Stevens, RG Ruth Ginsburg, DS David Souter, SB Stephen Breyer, SO Sandra O’Connor, AK Anthony Kennedy, WR William Rehnquist, AS Antonin Scalia and CT Clarence Thomas. (Online version in colour.)

If the independent model of equation (2.1) were correct, we would have $C_{ij}=0$ for all i≠j. Actually, the strong and positive correlations between the judges in the Second Rehnquist Court, shown in figure 1*a*, indicate that the judges generally vote together, even across ideological lines.

Correlation, however, does not directly indicate interaction or influence. Judges’ votes will be correlated owing to the facts of each case. For example, suppose each case before the court presented a simple circumstance with a well defined and uncontroversial legal solution. In any such case, either voting +1 is the correct thing to do, or voting −1 would be correct, but all judges should vote in the same way because of the facts of the case. If all cases were truly like this then one would expect to see many unanimous decisions, even if there were no interactions or influence between judges.

To make this a little more rigorous, we can imagine that each judge votes in the correct direction with a given probability. Suppose, for the sake of argument, that +1 were the correct direction for a particular case, and let us still assume the judges are independent. Then we can write the probability that judges vote $s=(s_{1},\ldots ,s_{n})$ in a general logit form

2.4 $$P_{+}(s|H)=\prod_{i=1}^{n}\frac{e^{Hs_{i}}}{e^{Hs_{i}}+e^{-Hs_{i}}},$$

where H parameterizes the log-odds, and a larger H means a higher tendency of voting +1. As mentioned, the assignment of +1 or −1 to each side of a case is arbitrary. If instead the correct thing to do on a particular case were to vote −1 we would have

2.5 $$P_{-}(s|H)=\prod_{i=1}^{n}\frac{e^{-Hs_{i}}}{e^{Hs_{i}}+e^{-Hs_{i}}}$$

(note the change to a minus sign in the numerator’s exponent). Given that we do not assume that we know the correct orientation, we should allow for either possibility with equal probability,

2.6 $$P(s|H)=\frac{1}{2}\left[P_{+}(s|H)+P_{-}(s|H)\right]=\frac{1}{2}\left[\prod_{i=1}^{n}\frac{e^{Hs_{i}}}{e^{Hs_{i}}+e^{-Hs_{i}}}+\prod_{i=1}^{n}\frac{e^{-Hs_{i}}}{e^{Hs_{i}}+e^{-Hs_{i}}}\right].$$

The votes in the model are not independent because the probability distribution does not factorize. Instead, they are conditionally independent. If we knew and could condition on the correct orientation, the votes would again be independent.^1^

The simple model of equation (2.6) has only one parameter, which represents the idea that any vote is decided by judges that share a common body of law and precedent, and the same facts are presented to each. There might be a relatively straightforward sense in which siding with either party is either correct or incorrect [20]. However, this can only account for a single global correlation between judges and not the rich pattern shown in figure 1.

To improve the model, we allow each individual judge to deviate from the global consensus at their own rate. Each judge i is given their own individual bias parameter $h_{i}$. In any particular case, these biases may either work in alignment or in opposition to the shared consensus force. This leads to four possibilities. Judge i may vote +1 with a log-odds of: $H+h_{i}$, $H-h_{i}$, $-H+h_{i}$ or $-H-h_{i}$. Since we do not assume we know the valence of each vote, the two outcomes of aligned and anti-aligned biases are equally likely and we have

2.7 $$P_{\mathrm{CI}}(s|H,h)=\frac{1}{2}\left(\frac{\cosh\left(\sum_{i=1}^{n}(H+h_{i})s_{i}\right)}{\prod_{i=1}^{n}2\cosh(H+h_{i})}+\frac{\cosh\left(\sum_{i=1}^{n}(H-h_{i})s_{i}\right)}{\prod_{i=1}^{n}2\cosh(H-h_{i})}\right).$$

The model described in equation (2.7) illustrates a simple model of n conditionally independent judges.^2^

Given dataset $D=\{s_{1},\ldots ,s_{K}\}$ with K cases, we can fit the model by applying Bayes’ Theorem to obtain the posterior probability distribution for model parameters [21]

2.8 $$P(h,H|D)=\frac{P(D|h,H)P(h,H)}{\int P(D|h,H)P(h,H)\;\text{d}h\;\text{d}H}\;,$$

where the likelihood of the dataset $P(D|h,H)=\prod_{k=1}^{K}P_{\text{I}}(s_{k}|h,H)$ is a product over the K data points indexed by k, the prior distribution over model parameters P(h,H) and the model evidence that integrates over the product of the two factors. We fix the prior distribution for the parameters to be a normal distribution with mean 0 and standard deviation 1/2.

Point estimates for parameters can be recovered by maximum *a posteriori* (MAP) estimates. These are found by maximizing equation (2.8) with respect to parameters. More generally, we can consider not just the optimal solution but the distribution around such a solution by estimating the entire posterior probability. Such an estimate also accounts for the model evidence P(D), which is the denominator in equation (2.8) (perhaps better known by its approximation, the Bayesian information criterion). Highly complex models are problematic because they can find seemingly good fits to any data. By contrast, a good model fits the real data well, but would not necessarily fit any possible counterfactual data well. The model evidence accounts for complexity by integrating over all possible parameter combinations [21–23]. Models with larger model evidence should be preferred, regardless of the number of parameters. We show our result for the conditionally independent model of the Rehnquist Court in table 1 and for the Roberts Court in table 2.

**Table 1.** Model evidence for the Second Rehnquist Court (larger is better). The model that incorporates both bias and interaction is better supported by the data than either bias or interaction alone. We adopt normal priors with mean 0 and standard deviation 1/2 for all parameters.

model	model evidence, log⁡P(D)
bias, (2.7)	−3481.34
interaction only, (2.9)	−3355.08
combined, (2.10)	−3304.20

**Table 2.** Model evidence for the Roberts Court (larger is better). The model that incorporates both bias and interaction is better supported by the data than either bias or interaction alone.

model	model evidence, log⁡P(D)
bias, (2.7)	−483.59
interaction, (2.9)	−458.89
combined, (2.10)	−440.65

Importantly, Bayesian methods allow us to account for uncertainty and balance models for their complexity.

**Figure 2.**
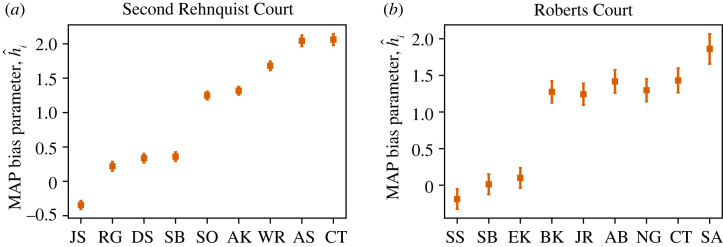
Best-fit parameters of the conditional independent model, equation (2.7). Justices are ordered from left to right by their Martin–Quinn ideology scores [19]. For both courts, the parameters from conditional independent model broadly agree with the ideological rankings. Error bars show one standard deviation in the posterior distribution. (Online version in colour.)

We summarize the model fit in figures 1 and 2. The MAP model parameters return a good fit to data statistics, capturing many of the pairwise correlations. The model also roughly matches the frequency of each observed voting outcome, figure 1*c*. This is perhaps surprising because in the model, the individual behaviour of each judge is dictated by only a single parameter, and all judges are conditionally independent. While our best-fit parameters ${\hat{h}}_{i}$ shown in figure 2 reveal an ideological dimension in the spirit of issue-space models [19,24,25], our model has the advantage of providing a clear probabilistic interpretation of the biases h; here, a unit increase is a factor of e increase in the probability. In addition, the model is significantly less complex, and we explicitly balance certainty from data and model complexity with Bayesian techniques. Taken together, these results indicate that two dimensions of hidden valence lead to a remarkably good, if incomplete, model for voting behaviour on the US Supreme Court.

### (b) Accounting for interaction

It is remarkable that a model of conditionally independent voters does so well. Justices decide on which cases to consider together (known as the ‘rule of four’ on the Supreme Court [3]), they sit on the same bench during oral arguments, they trade notes during the writing process [26], they strategize about opinion writing and assignment [27,28], and they are even friends [29]. The many examples of interaction suggest that there is ample space for them to influence each other. For example, judges switch, if rarely, their initially expressed conference vote before the final vote [30]. This suggests a fundamental ingredient is missing in the whole framework of conditionally independent models: judges interact.

We incorporate interactions into the model using a well-established model of pairwise interaction: the Ising model from statistical physics. This model choice can be justified by an appeal to the principle of maximum entropy [31], which says one should favour models that maximise Shannon entropy, given certain constraints. Constraining a model to fit pairwise correlations while maximizing Shannon entropy yields an exponential form

2.9 $$P(s|J)=\frac{1}{Z}\exp\left(\frac{1}{\sqrt{n}}\sum_{i<j}J_{ij}s_{i}s_{j}\right),$$

where Z is a constant that ensures that the distribution is normalized. Each parameter in the exponential $J_{ij}$ parameterizes the coupling between pairs of voters i and j. Since we are summing over all pairs of voters, positive contributions to the sum in the exponential increase the probability of observing an outcome s, whereas negative terms decrease the corresponding probability—just as in the logit model from equation (2.7). Then, a positive coupling, $J_{ij}>0$, indicates a tendency for a pair of voters to vote with one another, whereas a negative coupling, $J_{ij}<0$, indicates a tendency to vote against. This model has proved to be a remarkably accurate model of Supreme Court voting [17,32,33].^3^

So far, the model of equation (2.9) does not account for the valence of cases. Marginalizing over the unknown valence of the case as before, we obtain a model that combines both competing biases and interactions

2.10 $$\begin{array}{rl} P_{\text{C}}(s|J,H,h) & \;=\exp\left(\sum_{i<j}\frac{J_{ij}s_{i}s_{j}}{\sqrt{n}}\right)\left(\frac{\cosh(\sum_{i}(H+h_{i})s_{i})}{Z_{1}}+\frac{\cosh(\sum_{i}(H-h_{i})s_{i})}{Z_{2}}\right), \end{array}$$

with separate normalization terms $Z_{1}$ and $Z_{2}$. Note that the interactions through $J_{ij}$ do not depend on the valence, and so they factorize out. Furthermore, nested within equation (2.10) are two models: the conditional independence model when $J_{ij}=0$, and a solely interaction-based model when H=h=0 (the model of equation (2.9), studied in [17]). Hence, the combined model is more expressive than either and generalizes both.

After incorporating both fields and couplings we find a model that matches the data substantially better than the conditionally independent voters model. This increase in fit is not an artefact of the increased number of parameters of the model. When we compare models rigorously using the model evidence, we find that the model evidence significantly favours the combined model—with both biases and interaction—over a model with either only biases or only interactions on all natural courts with at least one hundred votes in the modern Supreme Court era. The results are listed for the two courts in tables 1 and 2.

Importantly, Bayesian model selection rigorously accounts for model complexity in way that is self-consistent. To demonstrate this, we performed model-fitting experiments on synthetic datasets. We sampled 100 sets of parameters from the priors of each model, and then generated synthetic datasets with 500 cases. For all 100 datasets in which the bias model of equation (2.7) was the true generative model, our method correctly identified it as the true model. Likewise, the interaction only model of equation (2.9) was correctly identified in all 100 cases. Finally, the full combined model of equation (2.10) was favoured in 71 out of 100 datasets in which is was the true model. In the other 29, a simpler model was preferred. The procedure thus correctly identifies the corresponding model on synthetic data most of the time and is conservative when it fails, underlining the separate contributions of bias and couplings in the courts.

For a more tangible assessment of model fit, we show in figure 1 model statistics for the most compelling fit, or the MAP model. In figure 1*b*, we compare the empirical covariance to those predicted by the models. The model captures the overwhelming tendency towards consensus displayed in fully positive correlations as well as the stronger tendencies to vote with one’s ideological bloc. It is also able to mimic the finer variation within the blocs including the emergence of a centrist bloc. In figure 1*c*, we show that the models accurately predict the entire probability distribution. A quantitative measure of this is the Bhattacharyya coefficient

2.11 $$B(P,Q)=\sum_{s}\sqrt{P(s)Q(s)}.$$

When B(P,Q)=1 the distributions P and Q are the same. We computed the Bhattacharyya coefficients for both models, compared to the empirical frequency distribution. The model that ignores interactions scores B=0.89, whereas with interactions we have B=0.96. These measures show that the combined model is not only optimal according to the model evidence but is highly accurate for nearly all observed voting patterns.

## 3. Analysis

### (a) Second Rehnquist Court

The model allows us to predict how the voters behave in the different contexts we have extracted, corresponding to when the biases H and h are aligned or anti-aligned. In other words, we can take the correct sign for some case to be +1 and the individual biases $h_{i}$ to be aligned with H or anti-aligned. Then, there are two possibilities, either

3.1 $$P_{+}(s|J,H,h)=\frac{\exp\left(\sum_{i=1}^{n}(H+h_{i})s_{i}+\sum_{i<j}J_{ij}s_{i}s_{j}/\sqrt{n}\right)}{Z_{1}}$$

or

3.2 $$P_{-}(s|J,H,h))=\frac{\exp\left(\sum_{i=1}^{n}(H-h_{i})s_{i}+\sum_{i<j}J_{ij}s_{i}s_{j}/\sqrt{n}\right)}{Z_{2}}.$$

Thus equations (3.1) and (3.2) translate the model biases that we recover each justice and turn them into expected conditional votes, roughly corresponding to whether the vote is a consensus or ideological vote.

**Figure 3.**
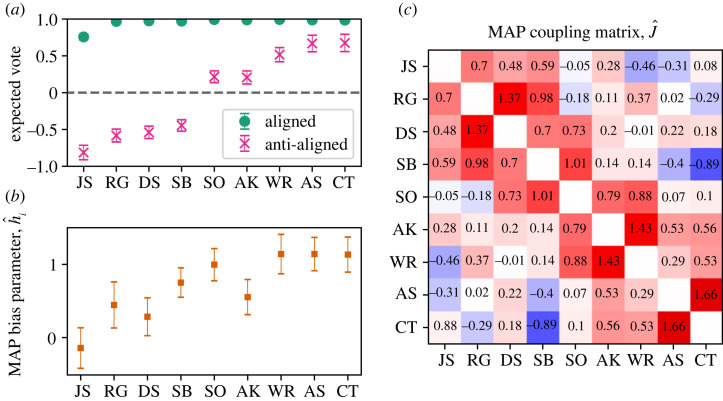
Model fits for the Second Rehnquist Court. (*a*) The expected vote of each judge, when the signs of H and h match and when they do not. This corresponds to a near-unanimous behaviour, or a 5-4 split. (*b*) Inferred most-likely values for the bias parameters, h. (*c*) Inferred most-likely values for the couplings, J. Variance of prior is 1/2 for the biases. Error bars show one standard deviation in the posterior distribution. (Online version in colour.)

When H and h are aligned as in equation (3.1), all justices are highly likely to vote +1 (equivalent to −1 if we flip the signs of both H and h) as we show with the green circles in figure 3*a*. This corresponds to a consensus dynamic where all judges vote similarly, although John Stevens’s (JS) maverick nature is also apparent since he deviates visibly from the others. In the anti-aligned case (red x’s), we see a 5-4 split on the signs of expected votes that corresponds to ideological division, and the ranking of voters along this axis is similar to the widely used liberal–conservative Martin–Quinn scores. This is also in agreement with the liberal–conservative labelling of the votes in the data from [13]. Thus, the combined effect of biases and couplings highlights individual voting statistics that align with two intuitive modes of behaviour, consensus and ideological division.

Notably, the expected votes in the anti-aligned scenario do not precisely track with the individual biases h in figure 3*b*. We find, for example, that the swing voter Kennedy (AK) and moderate liberal Souter (DS) to have much more liberal biases than their expected votes. Kennedy’s bias is quite surprising given that his expected vote in figure 3*a* is almost indistinguishable from O’Connor’s (SO). Furthermore, three conservatives Rehnquist (WR), Scalia (AS) and Thomas (CT) have almost indistinguishable ‘conservative’ biases that belie differences in their voting records. In the conditionally independent model fits, the only way a judge’s expected vote can be shifted is by changing the corresponding $h_{i}$; there is a very simple correspondence between bias and voting record (see figure 2 for comparison). Interactions complicate this picture. A justice may vote either more or less conservatively because of interactions with others. The difference between the results of the conditionally independent and the combined models suggests that inferences about individual-level biases can be obscured by the presence of interactions and unknown valence.

In fact, our model parameters suggest that the interactions play an important role in collective voting outcomes. In the aligned mode, the individual propensities for consensus are reinforced by the couplings. For example, the chances that Stevens (JS) dissents from a unanimous coalition is about 24% without the interactions, but once we account for the couplings this drops to 14%. The impact of couplings in driving the individuals to consensus implies that individuals’ patterns of voting are, at least in part, a response to their colleagues’ votes.

One way to see the effect of interactions is to embed the votes into a probability ‘landscape’ and to inspect its structure. We define a landscape by noting that every vote configuration is one vote-flip away from nine others because any of the nine justices could have voted differently. Any two configurations that differ by one vote can be thought of as being adjacent to one another. Along with this formulation of distance, the probability of each configuration then defines a landscape, where the peaks correspond to votes that are more likely than any of those near to them. By taking every voting configuration, calculating the voter who is most likely to flip, and iterating until we have hit a fixed point, we can determine the sets of votes out of the $2^{9}$ possible ones that all belong to the same set of peaks; this procedure clusters the votes according to the stable voting configurations to which they belong.

By following this procedure for the aligned biases and another for the anti-aligned case, we find that the two probability landscapes give different structures for the Rehnquist Court. Without interactions, only the biases determine the outcomes, forming a single peak in the probability landscape that are a unanimous vote and a 5-4 vote. In the case of aligned biases, the interactions sharpen the peak such that unanimity occurs an extraordinary 83% of the time, while the biases alone would only produce unanimity 48% of the time. In the case of anti-aligned biases, the interactions reshape the landscape, and we find several stable configurations for a 5-4 usual liberal–conservative split grouping together 45% of all $2^{9}$ voting configurations, an 8-1 with Stevens (JS) dissenting taking 25%, 9-0 unanimity taking 17% and a 7-2 with Scalia (AS) and Thomas (CT) dissenting taking 13%. Thus, interactions on the Second Rehnquist Court tend to strengthen the winning coalition, enlargen it, and recompose the majority in a way that obscures individual tendencies.^4^

### (b) Roberts Court

**Figure 4.**
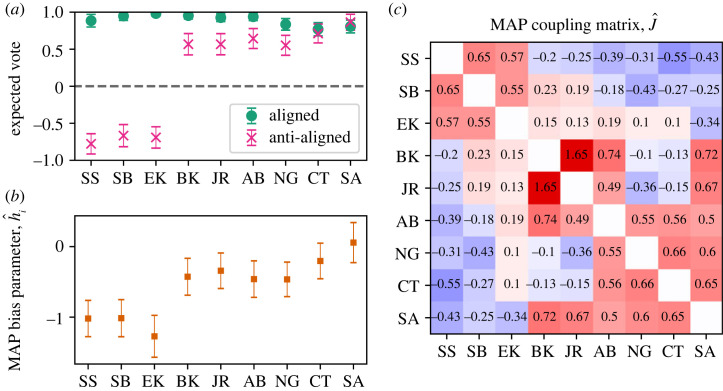
Model fits for the Roberts Court. (*a*) The expected vote of each judge, when the signs of H and h are aligned and when they do not. This corresponds to a near-unanimous behaviour and a 6-3 split. (*b*) Inferred most-likely values for the bias parameters, h. (*c*) Inferred most-likely values for the couplings, J. Interestingly, the strongest coupling by far is between Kavanaugh and Roberts. Judge initials SS for Sonia Sotomayor; SB, Stephen Breyer; EK, Elena Kagan; BK, Brett Kavanaugh; JR, John Roberts; AB, Amy Barrett; NG, Neil Gorsuch; CT, Clarence Thomas and SA, Samuel Alito. Error bars show one standard deviation in the posterior distribution. (Online version in colour.)

We again find strong evidence for the combined role of individual biases and interactions according to the model evidence in table 2. In figure 4, we show the model fit results.

The expected votes conditioned on the aligned and anti-aligned conditions reflect consensus and ideological division between left and right, where the three liberal justices are distinguished from the six conservative ones as we show with their expected votes in figure 4*a*. Again, the expressed voting tendencies disagree with their individual voting tendencies because of the interplay between bias and interactions.

Inspecting model parameters, we find qualitative similarities and differences from the Rehnquist Court. Inspecting the probability landscape, we again find a single peak in the aligned mode when the summed biases of the justices are all in the same direction, indicating one mode of voting with an overwhelming tendency to consensus.

In the anti-aligned case, the biases in figure 4*b* reveal a strong split, and none of the justices appear particularly close to the centre. Once we account for interactions, the probability landscape displays two maxima: the conservative-liberal 6-3 mode to which 52% of voting configurations belong and another, more surprising, peak which is a 5-4 mode with the liberals in the majority and joined by Kavanaugh (BT) and Roberts (JR). Unlike the Rehnquist Court, there is no peak at unanimity in the anti-aligned case. Thus, our results suggest that interactions, which seem to enhance the chances of consensus, are insufficient to overcome the partisan divide in the Roberts Court in contrast to the Rehnquist Court.

## 4. Discussion

We develop a minimal approach to collective models of voting that accounts for the multiple contexts in which individual biases may play a role. Using the US Supreme Court as an example and accounting for two different contexts, we discover an individual ‘ideological’ bias and a tendency to choose the ‘correct’ (i.e. consensus) outcome. With the model, we are able to capture higher-order, collective statistics in voting tendencies. Including interactions significantly improves the model with different inferred biases. This suggests interaction is an important confound in attempts to identify partisan alignment and caution is warranted for ideological measures that assume conditional independence.

The dominant and widely used approach to ideological measures are issue-space, ideal-point or spatial voting models. They have a rich history of development from the original application to political voting by Poole and Rosenthal and extensions have been applied to political voting more broadly [18,19]. The idea is to infer the space of issues in which votes are being cast and the locations of the voters in the space. The assumptions behind the models are that there is a low-dimensional issue space in which voters live (analogous to our contexts but more specific to the contents of the vote), voters have a utility function in that space, and they try to maximize their individual utilities. In the language of machine learning, the technique can also be described as a kernel function representation of voters [32]. On one hand, the approach is intuitive, is widely used, and the extracted issue dimensions lend themselves to partisan interpretation. On the other, the models are highly overparameterized, raising questions about parameter degeneracy and model complexity trade-offs; the interpretation of the issue space is subjective; and the approach assumes conditional independence of voters given the issue, which we show here is confounded by interactions. Thus, by revisiting the classic problem of inferring voter tendencies with a conceptually similar but minimal approach, we demonstrate how issue-space models could be made stronger.

In addition to accounting for interactions, a clear advantage of the model we propose is that it captures consensus, whereas issue-space models only capture the ideological division. Since between 1/3 and 1/2 of Supreme Court votes in the modern era are unanimous—an even larger fraction for appellate courts [12]—any model that misses such a substantial fraction of data performs poorly in comparison. Not only do our models fit the data well, but the combined model has 46 parameters for the nine voters, with which it captures nearly the entire probability distribution of votes (figure 1), or nearly all statistics one could compute on the data. In contrast with previous work, we make this argument concrete with a Bayesian framework to test whether or not the addition of interaction parameters is justified. The model evidence buttresses the success of the combined model; because it integrates over all parameter combinations, it is a confident indicator that the model balances accuracy with minimality.

The model in equation (2.10) reproduces the ideological divisions that we expected from previous work on the Rehnquist and Roberts Courts (figures 3 and 4). Yet, it shows ideological biases that are not proportional to the individual voting statistics. This means that ideological scores that do not account for interaction may be missing a crucial element and thus misleading about the internal preferences of justices.

More concretely, we predict how interactions may shape collective outcomes. When we inspect the probability landscape from the aligned context, we see that interactions grow the probability peak for unanimous outcomes.^5^ Interactions also shape new coalitions out of the partisan tendencies that one would expect from inspecting individual preferences in both the Rehnquist and Roberts Courts. In the Rehnquist Court, we find that interactions stabilize the usual 5-4 ideological split evident in the individual biases. They also overcome ideological biases to produce unanimity or form majorities that leave Stevens (JS) to dissent alone or push Scalia (AS) and Thomas (CT) into the minority. In the Roberts Court, we find the interactions stabilize the coalition of three most liberal justices with conservatives Roberts (JR) and Kavanaugh (BK). The emergence of these peaks as a result of the interactions indicate a quantitatively measurable role for interactions in building coalitions.

These results hold for the MAP landscape, which is the focus of the work, but the solutions can be degenerate: different parameter choices can lead to comparably good fits.^6^ The choice of prior may affect the solution landscape, and this points to the simple fact that is difficult to distinguish from correlations between shared biases and from interactions. Generally, we should be cautious when mapping model parameters to a mechanistic understanding of how decisions are reached and take seriously the uncertainties that come from the voting data. The strength of our minimal approach is that it is feasible to understand the range of uncertainty with Bayesian methods, whereas the calculations become increasingly difficult with more parameters.

Our work demonstrates how one might rely on voting statistics and minimal models to extract insights about voter tendencies while accounting for hidden valence and interactions. Beyond judicial voting, similar models motivated from the physics literature have been influential for systems ranging across examples from neural coding in biology to group decision-making [38–43]. Our approach develops a way to account for valence and model complexity in a framework that can go beyond binary voting, account for time, and generalize to other social and biological contexts, where the context of an action matters for the outcome.

## Data Availability

Code to reproduce all analyses, datasets and model parameters are available online on GitHub at https://github.com/gcant/CorrelatedVotingModel.

## Footnotes

1 Some readers may recognize this functional form as a restricted Boltzmann machine with one hidden unit.

2 Note, equation (2.7) has the form of a restricted Boltzmann machine with two hidden units. The connection can be made concrete because marginalization over the hidden contexts induces interesting correlations between the voters i and j. If the hidden contexts were real variables and were Gaussian distributed, we would have an exact mapping from the fields to the couplings $J_{ij}$ in (2.9): $J_{ij}=2\sqrt{n}(H^{2}+h_{i}h_{j})$. For the binary case we consider, such a simple relation cannot be found.

3 In general, the parameter J in equation (2.9) is a full-rank symmetric matrix. Since the diagonal elements of J have no effect, this leads to a model with (n2) parameters. Throughout this paper, we fit the general, full-rank model. However, low-rank versions of the model are also interesting. For example, if J is rank-1 then the model is exactly equivalent to a conditionally independent model arising from an external Gaussian field that couples voters together. We leave an exploration of the rank of J to future work.

4 The peaks of the probability landscape are the minima of the energy landscape in statistical physics. Hopfield proposed an interpretation of the minima $\xi^{\mu }$ as patterns indexed μ that neural systems try to memorize. When one writes the Hamiltonian for such a system, the couplings can be expressed in terms of the overlap between different memories. Thus, in this language, the orientations of the votes are akin to the search for optimal alignment with predisposed patterns and the basin around the energy minima as noisy versions around the minima. This can be related to the restricted Boltzmann machine, where the memories reflect the coupling to the hidden states [34,35].

5 This is a quantitative signal of the norm to consensus, which is an oft discussed aspect of the US Supreme Court [30,36,37]. Consensus is essential because the court has no constitutional mandate to enforce its laws but relies solely on perceived legitimacy, an important part of which is the size of the majority. In other words, they have an incentive to make decisions together rather than along slim margins. In the historical evolution of the court, this was the establishment of the single ‘opinion of the court’ from the assortment of *per curiam* opinions. A classic example of the recognition of the power of unanimity is the famous (and then quite controversial) decision to desegregate schools in Brown v. Board of Education. The tendency to consensus is also a feature of appeals courts, where anecdotally the small panels of judges are willing to work together to come to a shared decision [12].

6 One somewhat trivial case of degeneracy is that if we map H→−H and $h_{i}\to -h_{i}$, then model predictions are unchanged.
